# Supplementary material for: The Correlation of Tooth Sizes and Jaw Dimensions with Biological Sex and Stature in a Contemporary Central European Population
Source: Biology (Basel). 2024 Jul 28;13(8):569. doi: 10.3390/biology13080569 (PMC11351304; doi:10.3390/biology13080569)
Supplement: Supplementary file 1 [file biology-13-00569-s001.zip › biology-3064100-supplementary.pdf]

Supplementary Materials

Table S1. The correlations between the jaw dimensions. The effects of stature and sex are partial led out. Correlations above 0.5 are marked in dark grey and highlighted in bold. Correlations above 0.3 are marked in light grey.

Jaw dimension	1	2	3	4	5	6	7	8	9
1-Internal palatal length	1.00	-0.02	0.10	0.07	0.07	0.41	**0.79**	**0.68**	0.18
2-Palate end width	-0.02	1.00	**0.69**	0.38	0.28	0.27	0.28	0.09	**0.88**
3-Anterior palatal width	0.10	0.69	1.00	0.09	0.11	0.42	0.31	0.17	**0.68**
4-Palatal height	0.07	0.38	0.09	1.00	**0.60**	0.08	0.20	0.04	0.31
5-Anterior palatal height	0.07	0.28	0.11	**0.60**	1.00	0.04	0.11	0.05	0.15
6-Anterior mandibular width	0.41	0.27	0.42	0.08	0.04	1.00	0.46	0.46	0.46
7-Dental arch length of maxilla	**0.79**	0.28	0.31	0.20	0.11	0.46	1.00	**0.74**	0.47
8-Dental arch length of mandible	**0.68**	0.09	0.17	0.04	0.05	0.46	**0.74**	1.00	0.37
9-External dental arch width	0.18	**0.88**	**0.68**	0.31	0.15	0.46	0.47	0.37	1.00

Table S2. The loadings of the nine jaw dimensions on the two latent factors. Loadings above 0.5 are marked in dark grey and highlighted in bold. Loadings above 0.3 are marked in light grey.

Jaw dimension	1st latent factor	2nd latent factor
1-Internal palatal length	-0.029	**0.863**
2-Palate end width	**0.927**	-0.002
3-Anterior palatal width	**0.728**	0.124
4-Palatal height	0.235	0.064
5-Anterior palatal height	0.139	0.041
6-Anterior mandibular width	0.351	0.459
7-Dental arch length of maxilla	0.269	**0.867**
8-Dental arch length of mandible	0.130	**0.811**
9-External dental arch width	**0.909**	0.261
